# The Features of Checkpoint Receptor—Ligand Interaction in Cancer and the Therapeutic Effectiveness of Their Inhibition

**DOI:** 10.3390/biomedicines10092081

**Published:** 2022-08-25

**Authors:** Anna Kuzevanova, Natalya Apanovich, Danzan Mansorunov, Alexandra Korotaeva, Alexander Karpukhin

**Affiliations:** Research Centre for Medical Genetics, 1 Moskvorechye St., 115522 Moscow, Russia

**Keywords:** immune checkpoint, expression, therapy, target, immune response

## Abstract

To date, certain problems have been identified in cancer immunotherapy using the inhibition of immune checkpoints (ICs). Despite the excellent effect of cancer therapy in some cases when blocking the PD-L1 (programmed death-ligand 1) ligand and the immune cell receptors PD-1 (programmed cell death protein 1) and CTLA4 (cytotoxic T-lymphocyte-associated protein 4) with antibodies, the proportion of patients responding to such therapy is still far from desirable. This situation has stimulated the exploration of additional receptors and ligands as targets for immunotherapy. In our article, based on the analysis of the available data, the TIM-3 (T-cell immunoglobulin and mucin domain-3), LAG-3 (lymphocyte-activation gene 3), TIGIT (T-cell immunoreceptor with Ig and immunoreceptor tyrosine-based inhibitory motif (ITIM) domains), VISTA (V-domain Ig suppressor of T-cell activation), and BTLA (B- and T-lymphocyte attenuator) receptors and their ligands are comprehensively considered. Data on the relationship between receptor expression and the clinical characteristics of tumors are presented and are analyzed together with the results of preclinical and clinical studies on the therapeutic efficacy of their blocking. Such a comprehensive analysis makes it possible to assess the prospects of receptors of this series as targets for anticancer therapy. The expression of the LAG-3 receptor shows the most unambiguous relationship with the clinical characteristics of cancer. Its inhibition is the most effective of the analyzed series in terms of the antitumor response. The expression of TIGIT and BTLA correlates well with clinical characteristics and demonstrates antitumor efficacy in preclinical and clinical studies, which indicates their high promise as targets for anticancer therapy. At the same time, the relationship of VISTA and TIM-3 expression with the clinical characteristics of the tumor is contradictory, and the results on the antitumor effectiveness of their inhibition are inconsistent.

## 1. Introduction

Anticancer therapy based on the inhibition of immune checkpoints (ICs) is an actively developing field of study, and it has been widely used recently. Antibodies blocking immune checkpoints are used as therapeutics. The targeted checkpoints are mainly the PD-L1 (programmed death-ligand 1), expressed by the tumor, and the PD-1 (programmed cell death protein 1) and CTLA-4 (cytotoxic T-lymphocyte-associated protein 4) immune cell receptors. Along with the undoubted successes, some problems have also been discovered, including an insufficient number of patients responding to such therapy. To increase the effectiveness of therapy by blocking ICs, additional receptors and ligands are being investigated as targets of immunotherapy. By now, information on this topic has accumulated, the systematization and analysis of which can be useful both for understanding the current state of the problem and for updating the most promising areas of further research. Our article comprehensively examines a number of IC receptors. In addition to their description, data on their interaction with ligands and on the relationship of receptor expression with the clinical characteristics of tumors are also considered. These data were analyzed together with the results of preclinical and clinical studies of the therapeutic effectiveness of their blocking. The receptors TIM-3 (T-cell immunoglobulin and mucin domain-3), LAG-3 (lymphocyte-activation gene 3), TIGIT (T-cell immunoreceptor with Ig and immunoreceptor tyrosine-based inhibitory motif (ITIM) domains), VISTA (V-domain Ig suppressor of T-cell activation), and BTLA (B- and T-lymphocyte attenuator) are considered. These receptors and their ligands are currently being actively studied, and they all have several interacting molecules that can act as ligands. This situation complicates the interpretation of changes in antitumor immunity when inhibiting such receptors. However, an analysis of the relationship between their expression and the clinical characteristics of malignant tumors can provide additional information about their significance in the antitumor immune response. Overall, the comprehensive analysis carried out may allow us to more fully and accurately assess the prospects of the receptors of this series as targets of antitumor therapy. Although there are some reviews on ICs and their expression [1,2], the topic has not been considered in such a context or so comprehensively.

## 2. Immune Checkpoint Receptors

To obtain information, we performed a search on the PubMed, PMC, Omicsonline, and Embase databases using keywords. We also searched for information on the resource ClinicalTrials.gov (accessed on 10 June 2022).

Data on the clinical significance of the molecules considered in the work, as well as the results of preclinical studies, are presented in Table 1.

Data on current clinical trials utilizing the considered immune checkpoints are presented in Table 2.

### 2.1. TIM-3

TIM-3 is a transmembrane protein, expressed by T-cells, IFNγ-secreting T-regulatory cells (Treg), natural killer cells (NK cells), dendritic cells (DCs), macrophages, and mast cells [3]. TIM-3 is a receptor, an immune response regulator that ensures the formation of immunological tolerance and prevents the occurrence of autoimmune diseases by regulating the homeostasis of T-helper type 1 [4]. A decreased expression level of TIM-3 is associated with the development of diabetes and multiple sclerosis [5]. At the same time, the overexpression of Tim3 can contribute to the depletion of T-cells by limiting the pool of memory T-cells while enhancing the initial activation of T-cells and the generation of short-lived effector cells in acute and chronic infections [6]. In addition, the participation of TIM-3 in the activation of mast cells was revealed [7]. Increased TIM-3 expression by tumor-infiltrating lymphocytes (TILs) is indicated in many malignant neoplasms and is characteristic of effector lymphocytes with a depleted phenotype [8,9]. On the other hand, TIM-3 expression is characteristic of activated regulatory T-cells with immunosuppressive activity [10]. A significant role of TIM-3, expressed in APC and T-cells, in the regulation of CD8+ TILs trogocytosis in tumors has been shown. The use of mAb to TIM-3 is able to counteract the fratricidal process undergone by trogocytosed CD8+ T-cells [11].

#### 2.1.1. Interaction with Ligands

TIM-3 interacts with four ligands: phosphatidylserine, galectin-9, alarmin-1, and the CEACAM1 gene product.

Phosphotidylserine (a phospholipid that, when localized on the outer surface of the cell membrane, serves as a specific marker of apoptosis and a signal for phagocytic cells) binds to TIM-3 presented on DCs, triggering the processes of antigen cross-presentation and the subsequent elimination of apoptotic cells [12]. Given the low affinity for TIM-3 compared to that for TIM-1 and TIM-4, phosphatidylserine probably does not play a leading role in the regulatory mechanisms of T-cell homeostasis associated with TIM-3.

Data on the interaction of galectin-9 and TIM-3 are contradictory. Galectin-9 is involved in oncogenesis, cell transformation, cell cycle regulation, cell adhesion, and angiogenesis [13]. It was shown that galectin-9 binding to the TIM-3 molecule on the surface of T-helpers terminates the T-cell antitumor immune response [14]. TIM-3/galectin-9 interaction triggers a program of apoptotic death of CD4+ and CD8+ lymphocytes [15], which is suppressed when TIM-3 and PD-1 are co-expressed by lymphocytes [16]. On the other hand, resistance to anti-PD-1 antibody therapy has been observed in the presence of TIM-3+ lymphocytes and galectin-9-expressing myeloid-derived suppressor cells (MDSC) [17].

However, there is evidence that TIM-3 is not a galectin-9 receptor that alternatively binds to CD44 [18]. The predictive value of galectin-9 is also controversial. The co-expression of galectin-9 and TIM-3 has been detected in various types of cancer [14,19]. For a number of tumors, in particular for hepatocellular and colorectal carcinoma, the correlation of galectin-9 expression with better overall survival (OS), as well as with better progression-free survival (PFS) in gastric cancer (GC) and non-small cell lung cancer (NSCLC), was shown [20]. The article by Yang et al. provides opposing data [16].

The alarmin-1 protein (high-mobility group box 1, HMGB1), in addition to secretion by macrophages and monocytes, is secreted by dead tumor cells formed due to necrotic processes and therapeutic effects [21]. HMGB1 is known to stimulate the proliferation and differentiation of MDSC [22], in turn, inhibiting the activity of T-cells and NK cells and thus promoting the progression and metastasis of tumors [23]. It has been shown that the interaction of TIM-3 expressed on DCs with an alternative HMGB1 ligand, rather than galectin-9, suppresses innate immunity in the tumor microenvironment [24]. HMGB1 is associated with progression and metastasis in NSCLC [25] and colorectal cancer (CRC) [26].

According to meta-analysis, for a wide range of oncological diseases, the overexpression of HMGB1 correlates with a poor clinical prognosis [27]. It has been shown that the sensitivity of nasopharyngeal cancer cells to radio- and chemotherapy increases as a result of HMGB1 inhibition [28]. At the same time, HMGB1 increases the amount of CD8+ TILs [29] and also participates in the processes of tumor cell death through the activation of innate and adaptive antitumor immune reactions [30,31]. The consequences of the TIM-3/HMGB1 interaction in the context of carcinogenesis are poorly understood, and the diversity and complexity of the regulatory processes associated with HMGB1 require further study.

The fourth known TIM-3 ligand is CEACAM1, a cancer embryonic antigen (CEA) family glycoprotein. It is expressed by epithelial and endothelial cells, bone marrow cells, and immune cells, most intensively in response to their activation. CEACAM1 is involved in adhesion, phagocytosis, angiogenesis, proliferation, homeostasis, and immune regulation [32].

The binding of TIM-3 to the tumor cell-presented CEACAM1 suppresses T-cell function. The co-expression of CEACAM1 and TIM-3 is observed in tolerogenic CD4+ lymphocytes and depleted CD8+ TILs [33]. At the same time, being co-expressed in T-cells, TIM-3 and CEACAM1 are able to interact, forming a heterodimer, which activates the functions of TIM-3 by facilitating the maturation and localization of TIM-3 on the cell surface [34]. The inhibition of NK functions, in addition to the mechanism associated with TIM-3, occurs during the interaction of two CEACAM1 molecules localized on the activated NK and on the tumor cell [35]. A synergistic antitumor effect has been shown with the simultaneous blockade of TIM-3 and CEACAM1, as well as CEACAM1 and PD-L1, on murine colorectal cancer tumors [36].

Interestingly, functionally effective TIM-3-binding antibodies prevent TIM-3 from interacting with phosphatidylserine and CEACAM1, but not with galectin-9 [37]. On the other hand, data obtained by Linhares et at. refute the role of CEACAM1 as a ligand and activator of inhibitory functions of the TIM-3 receptor [38]. Thus, the modulating functions of CEACAM1 determine its significant effect on the processes of tumor immunoresistance; however, the inhibition of T-cell functions mediated by the interaction of CEACAM1 and TIM-3 requires further clarification.

The expression patterns of CAECAM1, which differ depending on the type of tumor cells and the stage of the disease, as well as the involvement of the protein in various cellular processes, complicate the understanding of the role of CEACAM1 in carcinogenesis. On cell lines of bladder cancer, both suppressive and stimulating action was shown due to the induction of angiogenesis. In the early stages of CRC, CEACAM1 inhibits tumor cell proliferation [34]. However, in advanced disease, CEACAM1 is highly expressed in some types of cancer and correlates with tumor progression. CEACAM1, a diagnostic and prognostic marker of melanoma, is found in tumor samples and sera from patients with pancreatic cancer (PC) and is overexpressed in advanced stages of CRC, NSCLC, and other cancers [39]. At the same time, in gastric cancer, an association of low CEACAM-1 expression with poor OS has been shown [40]. Thus, the feasibility of targeted therapy aimed at inhibiting the interaction of TIM-3 and CEACAM1 requires further study.

#### 2.1.2. Expression in Cancer

An increased expression of TIM-3 was detected in CD4+ and CD8+ TILs in lung cancer, stomach cancer, head and neck carcinomas, and melanoma [41] in antigen-specific T-cells of peripheral blood of patients with various types of cancer [5] and in tumor-infiltrating DCs compared to DCs in normal tissues [24]. A high level of TIM-3 expression by tumor-associated macrophages (TAMs) is associated with late stages of the disease and poor clinical prognosis in hepatocellular carcinoma (HCC) [42]. The effectiveness of the suppression of TIM-3 expression in relation to macrophage polarization and, as a consequence, the suppression of HCC cell growth have been shown.

TIM3 is often co-expressed with PD-1, and both proteins are markers of depleted and dysfunctional TILs [43,44]. TIM-3 expression by regulatory lymphocytes (CD4+) is correlated with disease progression in NSCLC, ovarian cancer (OC), prostate cancer (PC), and other types of cancer [3]. In colorectal cancer, the critical importance of TIM-3 in the progression of the disease has been shown [45]. TIM-3 is associated with progression and metastasis in cervical cancer and may serve as a prognostic marker [46].

#### 2.1.3. Preclinical Studies

The use of antibodies against TIM-3 stimulates the production of IFNγ, which enhances antitumor immunity. The antitumor efficacy of anti-TIM-3 is associated with the ratio of CD8+:CD4+ T-cells in the TILs pool. In tumor models, the combined use of antibodies targeting TIM-3, PD-1, and CTLA-4 has been shown to be more effective and well tolerated [47].

In models of lung adenocarcinoma, it was found that the use of antibodies targeting PD-1 can increase the expression of TIM-3, which explains the mechanism of the emergence of resistance to therapy. In this case, the effectiveness of the use of TIM-3 in overcoming resistance to therapy with antibodies targeting PD-1 has been shown [48]. In a study by Koyama S. et al., it was also noted that the expression of LAG-3 and CTLA-4 was increased on CD8+ T-lymphocytes bound by the used antibodies targeting TIM-3 and PD-1, which may reduce the effectiveness of therapy. The combined use of anti-TIM-3 and anti-CTLA-4 antibodies shows a synergistic effect in in vivo models [49].

#### 2.1.4. Current Clinical Trials

Several anti-TIM-3 antibodies are currently being tested in clinical trials for the treatment of hematologic tumors, solid tumors, and melanoma.

MBG453 (sabatolimab), a monoclonal anti-TIM-3 antibody, is being analyzed in several phase I and II clinical trials in various combinations with PD-1, TGFβ, P-selectin blockers, selective kinase inhibitors, and chemotherapy for the treatment of patients with myelofibrosis, myelodysplastic syndrome, leukemia, and advanced solid tumors [50]. The combination MBG453+azacytidine has reached phase III trials in patients with myelodysplastic syndrome or chronic myelomonocytic leukemia (NCT04266301). Results of phase I/II studies evaluating the safety and efficacy of MBG453 as monotherapy and in combination with spartalizumab (anti-PD-1) have been published (NCT02608268) [51].

TSR-022 is a monoclonal antibody targeting TIM-3. Four phase II and II clinical trials are currently registered. TSR-022 is used in combination with anti-PD-1 and chemotherapy (NCT03680508, NCT03307785, and NCT04139902) for the treatment of various solid tumors and melanoma. According to the results of a phase I clinical trial (NCT02817633), in the group of patients who received the TSR-022+TSR-042 (anti-PD-1) combination, the objective response rate (ORR) was 15% (3/20), and disease stabilization reached 40% (8/20) [52]. Research is ongoing.

The results of phase I clinical trials of the LY3321367 antibody used in combination therapy with anti-PD-L1 have been published [53].

A phase Ia/Ib clinical trial (NCT03752177) revealed the high immunogenicity of a bispecific antibody (LY3415244) to TIM-3 and PD-L1. The study was terminated, and the data have been published [54]. Phase I trials of the monoclonal antibody INCAGN02390 as monotherapy or in combination with inhibitors of PD-1, LAG-3, IDO1, or FGFR for solid tumors and melanoma therapy are ongoing.

Other TIM-3 inhibitors are also in phase I trials: the BGB-A425 antibody, which is being tested in malignant disease therapy in conjunction with anti-PD-1 (NCT03744468); the BMS-986258 antibody (NCT03446040); SHR-1702 (NCT04443751, NCT03871855); and the bispecific antibody RO7121661, binding PD-1 and TIM-3 (NCT04785820, NCT03708328) [55].

Thus, the use of antibodies against TIM-3 in cancer immunotherapy may have significant potential. However, the interaction of TIM-3 with a wide range of ligands and the participation of PD-1 in such interactions complicates the understanding of the results of its inhibition and leads to ambiguity in the relationship between TIM-3 expression and clinical characteristics. It is evidently necessary to study the totality of the immune components involved in interactions with TIM-3 in order to determine the combinations that achieve the desired effect of TIM-3 inhibition.

### 2.2. LAG-3

The LAG-3 gene (CD223) encodes a protein that negatively regulates the activation, proliferation, effector functions, and homeostasis of T-cells [56,57] and dendritic cells participating in preventing the development of autoimmune reactions in normal tissues [58] and regulating the immune response in chronic infections [59]. Due to the partial similarity of extracellular domains, LAG-3 and CD4 were presumably developed by gene duplication. However, differences in their intracellular domains result in their opposite functions [60]. The LAG-3 protein is presented in a transmembrane and soluble form (sLAG-3) formed by alternative splicing. It has been shown that under the action of ADAM10 and ADAM17 metalloproteases, the extracellular part of the receptor also passes into a soluble form [61]. LAG-3 is constitutively expressed by natural T-regulatory cells (Tr1), DCs, NK cells, and B-cells and is not found on naive T-cells; however, its expression is strongly increased after the activation of CD4+ and CD8+ lymphocytes, including TILs [62]. The modulating functions of LAG-3 correlate with the level of receptor expression [63]. The activation of LAG-3 reduces the production of various immunostimulatory interleukins (IL) and increases sensitivity to Treg signaling, thereby increasing T-cell tolerance and accelerating their depletion [62].

#### 2.2.1. Interaction with Ligands

Several molecules are known to interact with LAG-3. The MHC class II molecule (MHCII) is normally expressed on antigen-presenting cells (APCs) but is often present in tumor cells [64]. The tumor-specific expression of MHCII may contribute to tumor recognition by the immune system and, therefore, affect the effectiveness of antitumor immunity. MHCII is associated with survival, increased numbers of CD4+ and CD8+ T-cells in the TILs, and a good response to anti-PD-1 and PD-L1 immunotherapy in some cancers [65]. MHCII acts as a ligand for both CD4 and LAG-3, with a much greater affinity for the latter. Therefore, it was assumed that LAG-3, at a high level of expression, effectively competes for binding to MHCII, thus blocking the stimulatory effect of CD4. However, Maruhashi T. et al. showed the presence of a mechanism of inhibition of CD4+ T-cells based on the preferential binding of the LAG-3 receptor to a conformational stable peptide–MHCII complex (pMHCII). In addition, it has been shown that the functions of LAG-3 depend on the presence of its intracellular domain, and the obstruction of CD4 binding to MHCII is not the determining mechanism of the negative immunomodulation of T-cell activity [66]. In general, the therapeutic significance of the mechanisms based on the LAG-3/MHCII interaction requires further investigation.

Fibrinogen-like protein (FGL-1), a protein secreted by liver cells, is highly expressed in tumors that are associated with a poor clinical prognosis. Elevated plasma levels of FGL-1 in cancer patients are associated not only with poor prognosis, but also with resistance to anti-PD-1/PD-L1 therapy [67]. The role of FGL-1 in invasion and metastasis in gastric cancer is known [68]. The FGL-1/LAG3 interaction appears to be an important, MHCII-independent, alternative mechanism for tumor evasion from immune defenses. Models show that FGL-1 inhibits the antigen-specific activation of CD8+ T-cells and the blockade of FGL-1/LAG-3 interaction stimulates tumor immunity [67]. The reduced expression level of FGL-1 also increases the efficiency of CD8+ T-cell activation during LAG-3 blockade [69]. Further study of the mechanisms of LAG-3 activation associated with FGL-1 will allow us to assess the prospects for the therapeutic use of the ligand.

There are many mechanisms of homeostasis of T-cell regulation coupled with galectin-3. For example, the regulation of T-cell receptor activation is closely coupled to galectin-3, and the binding of galectin-3 to CD45 induces T-cell apoptosis [70]. Galectin-3 also causes the suppression of cytotoxic T-lymphocyte functions as a LAG-3 ligand [71]. The restoration of cytolytic functions of CD8+ T-cells in response to the inhibition of galectin-3 was shown, which indicates the role of galectin-3 in the suppression of antitumor immunity. The direct involvement of galectin-3 in the processes of metastasis was revealed [72,73,74], as well as the association of galectin-3 expression with poor clinical prognosis [75]. However, in some malignant diseases, in particular melanoma and glioblastoma, the presence of galectin-3 is beneficial for patients [76]. Due to the diversity of functions and the wide distribution of galectin-3 in various tissues, its use as a target molecule, together with other agents, can be an effective approach in the treatment of oncological diseases.

LSECtin is a type II transmembrane protein that is mainly expressed in the liver. Models have been used to show the role of LSECtin in stimulating tumor development by activating the BTN3A3 receptor [77]. A high level of soluble LSECtin in the blood serum of patients with colon carcinoma is associated with the presence of liver metastases [78]. The expression of LSECtin and its interaction with the LAG-3 molecule are shown on B16 melanoma cells. It is accompanied by the suppression of the T-cell antitumor response, and the blockade of LSECtin/LAG-3 interaction restores the secretion of IFNγ [79]. Generally, the role of LSECtin in the development of cancer is poorly understood.

#### 2.2.2. Expression in Cancer and Preclinical Studies

LAG-3 overexpression in TILs and Tregs in patients’ peripheral blood is associated with T-cell depletion, as well as with tumor progression and poor clinical prognosis in many types of solid and hematological neoplasms [60].

The increased expression of LAG-3 and PD-1 in TILs has been established on renal cell carcinoma (RCC). It has been shown that the therapeutic use of PD-1 leads to an increase in the expression level of LAG-3 [80]. In NSCLC, the co-expression of LAG-3 and PD-1 on TILs and PD-L1 in tumor cells is shown [81]. A synergistic effect was observed from the combined use of antibodies targeting LAG-3 and PD-1 in various models of mouse tumors [82].

A recent study of patients with uveal melanoma (UM) showed a correlation between LAG-3 gene expression and its ligands galectin-3, LSECtin, and HLA class II in high-risk UM (all *p* ≤ 0.001) [83]. Thus, LAG-3 is a good candidate for targeted therapy for a wide range of cancers. The observed co-expression of LAG-3 and PD-1 and the high therapeutic efficacy of the simultaneous use of several antibodies shown in models formed the basis of clinical trial schemes.

#### 2.2.3. Current Clinical Trials

LAG-3 antagonists are being actively developed and tested [84]. Below are the main data resulting from some of these tests.

The LAG-3 Ig fusion protein is a highly potent activator of antigen-presenting cells. Clinical trials in patients with pancreatic carcinoma showed good tolerability of therapy with IMP321, as well as the combination of IMP321 + gemcitabine [85]. In combination therapy using IMP321 with paclitaxel, an objective response was observed in 50% of patients with metastatic breast cancer (MBC) [86]. IMP321 is currently being used as monotherapy and as part of combination therapy with chemotherapy and PD-1 inhibitors in several phase I and II trials.

Favezelimab (MK-4280) is an anti-LAG-3 antibody. At the initial stages of phase I/II clinical trials (NCT02720068), in the group of patients receiving monotherapy with MK-4280, a partial response was observed in one patient, and in the group receiving combination therapy with MK-4280 + pembrolizumab, a partial response was seen in 15 patients. Stabilization of the disease was achieved in 17% and 40% of cases, respectively [87]. MK-4280 is registered in several other clinical trials for the treatment of hematological neoplasms (NCT03598608), advanced solid tumors (NCT02720068) and advanced NSCLC (NCT03516981).

Relatlimab (BMS-986016) is a monoclonal antibody that blocks the interaction of LAG-3 and MHC-II in tumor cells [88]. Several studies are underway on BMS-986016 in combination with Nivolumab (NCT03743766, NCT04552223, NCT03623854, and NCT04913922). Based on interim clinical trial results (NCT01968109) of BMS-986016 in a cohort of patients (68) with melanoma, an overall response rate of 11.5% and a disease control rate of 49% were achieved among patients who progressed despite prior anti-PD-1/PD-L1 therapy [89]. In another phase II trial (NCT03470922), the median PFS was 10.1 months (95% confidence interval [CI], 6.4 to 15.7) with relatlimab–nivolumab as compared with 4.6 months (95% CI, 3.4 to 5.6) with nivolumab (hazard ratio for progression or death, 0.75 [95% CI, 0.62 to 0.92]; *p* = 0.006 by the log-rank test). PFS at 12 months was 47.7% (95% CI, 41.8 to 53.2) with relatlimab–nivolumab as compared with 36.0% (95% CI, 30.5 to 41.6) with nivolumab [90]. In a phase I/II randomized trial for patients with relapsed refractory multiple myeloma (NCT04150965), the immunological effects and safety of BMS-986016 and BMS-986207 (anti-TIGIT) are being evaluated.

A number of studies on in vivo models show a synergistic effect of a combination of antibodies (TSR-033, REGN3767) against LAG-3 and PD-1 (cemiplimab) [91,92]. At the same time, according to E. Burova et al., the antitumor effect largely depends on the dose of cemiplimab. The TSR-033 antibody is currently being tested in two clinical trials for the treatment of solid tumors (NCT02817633, NCT03250832).

Antibody LAG525 (ieramilimab) is used both as a monotherapy and in combination with anti-PD-1 for advanced tumors. In the published results of the study NCT02460224, the combination of ieramilimab with spartalizumab (*n* = 121) showed antitumor efficacy against advanced/metastatic solid tumors with 3 (2%) complete responses and 10 (8%) partial responses [93]. ClinicalTrials.gov also provides results of NCT03365791, NCT03499899 studies. The evaluation of LAG525 for BC (NCT03742349) and melanoma (NCT03484923) therapy is ongoing.

Bispecific antibodies are also being actively developed and tested. Preclinical studies of FS118, a bispecific antibody to LAG-3 and PD-L1, showed greater binding activity, T-cell enhancement efficiency, and antitumor efficacy compared to the combination of the corresponding antibodies [94].

RO7247669 is a bispecific antibody that binds LAG-3 and PD-1. Preclinical trials on models of pancreatic carcinoma have shown the high efficiency of therapy with bispecific antibodies, and the complete suppression of tumors. RO7247669 is undergoing clinical trials in patients with metastatic solid tumors (NCT04140500, NCT04785820, and NCT05419388) [95].

Therefore, the available preclinical and clinical data indicate that LAG-3 inhibition holds promise for stimulating the immune response to cancer, especially in combination with anti-PD-L1.

### 2.3. TIGIT

TIGIT is a co-inhibitory receptor, expressed by all types of T-lymphocytes, as well as NK cells [96]. The receptor is involved in maintaining self-tolerance. The positive effect of TIGIT in regenerative hyperplasia was revealed: the absence of the receptor impairs liver regeneration in vivo [97].

Several immunoregulatory mechanisms involving TIGIT have been described to date. The interaction of TIGIT with the ligand causes the phosphorylation of its cytoplasmic domain, which triggers processes that block the transmission of intracellular signals along the PI3K and MAPK pathways and the activation of NF-κB, which, in turn, leads to the suppression of the cytotoxic functions of NK cells [98]. In addition, the interaction of this receptor with the ligand leads to the phosphorylation of the latter and the triggering of modulating signals in DCs [99]. TIGIT has been reported to directly inhibit T-cell proliferation and effector functions by downregulating T-cell receptor (TCR) and activating CD28 signaling [100].

#### 2.3.1. Interaction with Ligands

Many ligands are known for TIGIT, including PVR (CD155), nectin-2 (CD112), and nectin-4 (PVRL4) [101].

The main TIGIT ligand is CD155 (poliovirus receptor, PVR), constitutively expressed by DCs, T- and B-cells, and cells of various types of healthy tissues. CD155, in addition to TIGIT, interacts with the activating receptor DNAM-1 (CD226) and the CD96 protein; however, it has a greater affinity for TIGIT, which is responsible for the significant role of CD155 in the induction of inhibitory immune signals [102]. The formation of a pair of TIGIT with PVR present on the DC membrane leads to the transformation of the latter and the acquisition of tolerogenic properties, which is characterized by a decrease in the secretion of IL-12 and an increase in the secretion of IL-10 [100].

In addition to participating in the regulation of immune cell functions, CD155 is involved in the processes of adhesion and migration, and models show the importance of CD155 in the survival and proliferation of cancer cells [103,104,105]. Overexpression in a number of tumors and the presence of a soluble form of CD155 in the blood serum of patients is associated with a poor clinical prognosis [102,106,107]. The association of the co-expression of TIGIT and CD155 with an unfavorable disease course in lung adenocarcinoma has been shown [108] and with primary squamous cell carcinoma of the esophagus [106].

It has been shown that TIGIT is able to interact with the complementary co-stimulatory receptor CD226, disrupting the formation of CD226 homodimers, which suppresses the function of this protein [109]. In addition, TIGIT, having a higher affinity for CD155, competes with DNAM-1 for binding to CD155, negatively modulating T-cell functions [110].

Hematopoietic cells express nectin-2 (CD112). Interaction with TIGIT or DNAM-1 leads, as in the case of CD155, to the corresponding transmission of inhibitory or stimulatory signals to immune cells. Nectin-2 is expressed in breast and ovarian tumors [111]. Having a lower affinity for TIGIT compared to CD155, nectin-2 was not considered as a therapeutic target. However, paired with the recently discovered PVRIG inhibitory receptor, nectin-2 is of interest for further research [112].

Recently, the protein nectin-4 (PVRL4) has been reported as a new TIGIT ligand that does not interact with CD226 or CD96, unlike other inhibitory receptor ligands. Nectin-4--blocking antibodies stimulate an NK-mediated antitumor response [113]. The participation of nectin-4 in the processes of proliferation, invasion, and metastasis through the activation of Pi3k/Akt and WNT/β-catenin signaling pathways has been shown in models of breast cancer cell lines [114]. The revealed hyperexpression of nectin-4 by tumor tissues of various malignant neoplasms is associated with tumor aggressiveness and poor clinical prognosis [115,116]. The available data allow us to consider nectin-4 as a promising therapeutic target for a wide range of oncological diseases.

Thus, the available data confirm a significant negative impact of the processes induced by TIGIT ligation on the effectiveness of the antitumor immune response and the course of cancers. At the same time, the development of effective therapeutic approaches requires an understanding of multiple interactions and the complexity of signal modulation of the system under consideration.

#### 2.3.2. Expression in Cancer and Preclinical Studies

Kurtulus et al. revealed TIGIT expression in natural regulatory T-cells (TIGIT+ FOXP3+ nTregs). It has been shown that intracellular TIGIT signaling contributes to the maintenance of TIGIT+ FOXP3+ nTreg homeostasis, which, in turn, suppresses the antitumor functions of effector T-cells [117]. TIGIT overexpression in TILs is associated with metastasis and poor clinical prognosis in gastric cancer and melanoma [118,119]. A high ratio of TIGIT/DNAM-1 to Treg in the TIL pool is associated with a poor clinical prognosis in patients treated with antibodies targeting PD-1 and/or CTLA-4. The co-expression of TIGIT with inhibitory receptors LAG-3, TIM-3, and PD-1 was revealed [120,121].

Increased expression of the TIGIT receptor was found in tumor-infiltrating NK cells relative to that in NK cells in peripheral blood and peritumoral zones. In addition, the overexpression of TIGIT on NK cells from patient tumors is associated with the presence of invasions into the lymph nodes [122]. In CRC with microsatellite instability (dMMR), an increased frequency of TIGIT overexpression in tumor tissues has been shown among patients with advanced disease, and TIGIT expression is associated with a decrease in median disease-free survival [123]. Similar results were obtained in other works [121,123].

The blockade of TIGIT has been shown to prevent the depletion of NK cells and stimulate NK-mediated tumor immunity, activate antitumor T-cell immunity, and promote the formation of immune memory in models of tumor retransplantation [122]. In several studies using in vivo models, the co-inhibition of TIGIT and PD-1 or PD-L1 with antibodies exhibited a significant therapeutic effect, up to the complete elimination of tumors [122,124,125]. In response to therapy with antibodies targeting PD-1, an increase in the expression of TIGIT ligands by tumor cells has been shown [126], which probably reflects adaptive processes in tumor cells and explains the high antitumor efficacy of the joint use of blocking antibodies.

Preillon et al., studying the actions of antagonistic antibody EOS-448 against TIGIT, showed several mechanisms that ensure the antitumor efficacy of specific proteins, namely the restoration of the functions of effector T-cells; the induction of antibody-dependent cellular cytotoxicity against regulatory T-cells, due to the high expression of TIGIT by cells of this subtype; and a direct cytotoxic effect on TIGIT+ tumor cells in cases of hematological diseases [127]. In vivo models show the high efficiency of the combined inhibition of PD-1 and CD96 or TIGIT and CD96, as well as the better tolerability of such combinations in comparison with anti-PD-1 and CTLA-4 therapy [128]. In preclinical studies, COM902, a monoclonal antibody targeting TIGIT, has been shown to enhance antitumor immune response and suppress tumor growth [126]. TIGIT-blocking antibodies have been shown to be effective in enhancing NK-mediated antitumor immunity [129]. Ex vivo models have demonstrated the effectiveness of the joint inhibition of TIGIT and PD-1 in relation to the restoration of the functions of CD8+ T lymphocytes derived from the TILs population of hepatocellular carcinoma [130].

#### 2.3.3. Current Clinical Trials

Vibostolimab (MK-7684), a monoclonal antibody targeting TIGIT (NCT04165070, NCT04305054, NCT04305041, NCT02964013, NCT04303169, NCT05007106, NCT05005442, NCT05226598), is undergoing phase I and II clinical trials, used both as a monotherapy, or in combination with PD-1 inhibitors, and in chemotherapy in patients with metastatic solid tumors and melanoma. The results of the study NCT02964013 have been published. In part A, in the group of patients (34) treated with vibostolimab alone, 56% of patients had treatment-related adverse events (TRAEs), whereas in the group receiving the combination of vibostolimab with pembrolizumab, this figure rose to 62% of patients. Grade 3–4 TRAE occurred in 9% and 17% of patients, respectively. No dose-limiting toxicity was reported. The confirmed objective response rates (ORR) were 0% for monotherapy and 7% for combination therapy. In part B, 39 patients had anti-PD-1/PD-L1 naive NSCLC, and all received combination therapy. TRAEs occurred in 85% of patients. The confirmed ORR was 26%, with responses observed in both PD-L1-positive and PD-L1-negative tumors. In a group of 67 patients with anti-PD-1/PD-L1-refractory NSCLC (monotherapy, 34; combination therapy, 33), the most commonly reported AEs were rash and fatigue (21% each) with monotherapy and pruritus (36%) and fatigue (24%) with combination therapy; the confirmed ORR was 3% for monotherapy and 3% for combination therapy [131].

Etigilimab (OMP-313M32), an antibody targeting TIGIT, is undergoing a phase II clinical trial for the treatment of platinum-resistant recurrent clear cell ovarian, primary peritoneal, or fallopian tube cancer in combination with nivolumab (NCT05026606). 313R12, an etigilimab analog, has been shown to be effective against colon and kidney tumors and melanoma in preclinical trials [132]. A phase Ia/Ib clinical trial (NCT03119428) showed an acceptable safety profile of etigilimab, both as a monotherapy and in combination with nivolumab [133].

Antibody BMS-986207 is being tested in phase I and II clinical trials, for the treatment of solid tumors as monotherapy (NCT02913313) or in combination with nivolumab (NCT04570839, NCT05005273), in melanomas alone or in combination with anti-LAG3 (NCT04150965).

Tiragolumab in clinical trials (NCT02864992) has shown its effectiveness in the treatment of patients with NSCLC carrying mutations in the MET gene. Approximately 50% of cases achieve a partial response [134]. In another clinical trial, the combination of tiragolumab + atezolizumab (anti-PD-L1) compared with the combination of placebo + atezolizumab for the treatment of NSCLC also achieved an increase in ORR (37.3% and 20.6%, respectively) and median disease-free survival (5.6 and 3.9 months, respectively) [135]. Currently, for the treatment of a wide range of solid tumors, tiragolumab is included in several dozen phase I-III clinical trials.

Other anti-TIGIT antibodies (domvanalimab (AB154) and ASP8374) are also in various stages of clinical trials [136,137].

Thus, the use of TIGIT as a target molecule is a promising strategy for neoplasm therapy, especially in combination with the inhibition of other immune cell receptors.

### 2.4. VISTA

VISTA or PD-1H (programmed death-1 homolog) is predominantly expressed by myeloid cells, as well as by CD4+ and Foxp3+ T-regulatory cells [138]. Studies of VISTA expression in cancer diseases have shown the presence of protein on TILs and macrophages and its absence on cells of most types of tumors [139]. However, in a number of studies, the expression of VISTA by tumor cells was detected in different proportions of samples in NSCLC [140], hepatocellular carcinoma [141], ovarian and endometrial cancer [142], melanoma, stomach cancer, and breast cancer [143]. VISTA negatively regulates T-cell activation, proliferation, and cytokine production [144] and specifically suppresses the immune response mediated by CD4+ T-cells [145]. However, in a study by Mercier et al., the suppression of lymphocyte functions was mediated by the activation of cell receptors by a fusion protein (VISTA-Ig) acting as a ligand [146]. On the other hand, the increased proliferation and production of VISTA−/− cytokines by CD4+ T-cells indicates VISTA receptor function [145]. In addition, VISTA directly regulates the effector functions of myeloid cells [147]. Thus, understanding the complex functioning of VISTA requires a detailed study of the associated immune regulatory mechanisms.

#### 2.4.1. Interaction with Ligands

According to existing data, the VSIG-3 protein (V-set and immunoglobulin domain containing 3), also called IGSF11 (immunoglobulin superfamily 11), acts as one of the VISTA ligands. The involvement of VSIG-3 in cell adhesion processes has been shown on human tumor cell lines [148]. Its expression is found mainly in the tissues of the testes and ovaries and is found to a lesser extent in the tissues of the brain and kidney [149]. VISTA/VSIG-3 interaction in vitro suppresses the production of IL-2 and IFNγ cytokines by CD3-activated T-cells, as well as the secretion of CCL5, CCL3, and CXCL-11 chemokines by peripheral blood mononuclear cells [147,150].

The expression of VSIG-3 by tumor tissues was found in colorectal and hepatocellular cancers, as well as in intestinal-type GC [151]. In a recent work by Ghouzlani et al. [152], conducted on samples obtained from patients with glioblastomas, it was revealed that the overexpression of VSIG-3 is associated with the expression of VISTA, as well as with PD-L1 and PD-1, with a high degree of tumor malignancy and a poor clinical prognosis.

The available data suggest an immunosuppressive role of VSIG-3; however, to date, the mechanisms of action of the VSIG-3 protein in the context of the pathogenesis of malignant neoplasms are poorly understood, as is its significance. Interestingly, in the work of Johnston et.al. discussed below, no specific interaction of VISTA/VSIG-3 was found [153].

A specific interaction putatively inhibiting antitumor cytotoxicity by triggering immune cell apoptosis was found between VISTA and galectin-9 [154]. Galectin-9 is also a ligand for several other molecules, including the inhibitory T-cell receptor TIM-3. The analysis of NSCLC tumor material revealed the expression of galectin-9 by both tumor cells and TILs [155]. The study of samples from patients with peritoneal carcinomatosis showed a high level of expression of galectin-9, VISTA, and TIM-3-depleted TILs [156]. In general, the role of galectin-9 in the regulatory mechanisms associated with VISTA is poorly understood.

The cell adhesion molecule PSGL-1 (P-selectin glycoprotein ligand-1) is detected in all cells of the myeloid and lymphoid series and is represented by two forms characteristic of different types of hematopoietic cells [157]. PSGL-1 is a receptor for a wide range of molecules: P-, L- and E-selectins; chemokines; Siglec-5 protein; and versican. PSGL-1 is involved in immune cell migration and the regulation of monocytes and affects the progression of tumors [158,159]. Johnston et al. demonstrated the ability of PSGL-1 to bind to VISTA at acidic values of the medium (pH 6.0), which are more characteristic of the tumor microenvironment. At lower pH values, an enhanced inhibitory effect of VISTA was shown, and the use of antibodies capable of blocking the VISTA/PSGL-1 interaction in vivo restored the proliferative and secretory functions of T-cells [153].

The consideration of VISTA as a therapeutic target requires a deep study of the complex processes associated with the work of this protein. The ability of VISTA to modulate the functions of cells of the lymphoid and myeloid lineage and the expression of VISTA/PSGL-1/VSIG3/galectin-9 molecules in a wide range of cells of the tumor microenvironment, as well as data on the presence of specific modulations to the immune response with the participation of VISTA in the context of the selective role of pH, expands the possibilities of finding and developing effective immunotherapeutic drugs.

#### 2.4.2. Expression in Cancer

The study of expression in tumor tissues revealed the ambiguity of the influence of the VISTA molecule from prognostic and therapeutic points of view. VISTA expression correlated with improved overall survival in pT1/2 esophageal adenocarcinoma [160], HCC [141], and ovarian cancer [161]; 5-year survival for NSCLC [162]; and disease-free survival for estrogen-receptor-negative, progesterone-receptor-negative, and invasive ductal carcinoma [163]. At the same time, VISTA expression is associated with the epithelial–mesenchymal phenotype of tumor microenvironment cells [164], with poor overall survival among patients with oral squamous cell carcinoma [165] and pancreatic cancer [166]. In another study, there was no association between VISTA expression in pancreatic tumors and survival rates [167]. An inverse relationship was shown between the level of VISTA expression in infiltrating myeloid cells and their number, as well as the cytolytic functions of CD8+ TILs in renal cell carcinoma [168]. An analysis of 464 samples of GC showed that the increased expression of VISTA by both tumor cells and immune cells is more characteristic of intestinal tumors. In addition, an association between VISTA and PD-L1 expression was found. There was no association between VISTA expression and disease stage or metastasis [169]. However, the authors noted an increase in the number of infiltrating immune cells highly expressing VISTA in gastric cancer samples from stage T1 to stage T2 and a decrease from stage T2 to T3. In prostate cancer and melanoma, patients treated with ipilimumab (anti-CTLA-4) showed an increase in TILs and VISTA-expressing M2 immunosuppressive macrophages [170].

#### 2.4.3. Preclinical Trials

In response to blocking VISTA with the use of antibodies in model experiments, an increase in the number of TILs and the restoration of the functions of CD8+ T-cells were observed [147]. In another experiment, an increase in the expression of chemokines (CXCL9/10, CCL4/5), which is significant for lymphocyte recruitment, as well as cytokines (IFNβ, IL6, IL12, IL23, IL27, TNFα) stimulating the antitumor T-cell response, was observed in tumor tissues [144]. However, the effective suppression of tumor growth was observed only when anti-VISTA antibodies were used in combination with anti-PD-1 antibodies [171] or CTLA-4 [172].

In in vivo models with VISTA blockade, an increase in tumor infiltration by immune cells and a decrease in the number of myeloid suppressor cells (MSCs) were observed. The antitumor efficacy and positive effect on survival of the use of a combination of anti-VISTA and anti-PD-1 antibodies has been shown [173]. The therapeutic effect of anti-VISTA antibodies has been demonstrated in OC models highly expressing VISTA [143].

Experimental in vivo models show the antitumor efficacy of the SG7 antibody, which inhibits VISTA binding to VSIG-3 and PSGL-1. Interestingly, when using two variants of antibodies (activating FcR and, accordingly, triggering intracellular processes of the depletion of immune cells or blocking ligation), a comparable suppression of tumor growth was obtained [174].

Studies of the HMBD-002 antibody showed a high antitumor effect and no toxicity. The initiation of Phase I clinical trials was announced in 2021 [175].

#### 2.4.4. Clinical Trials

Currently, according to the database Clinicaltrial.gov, one phase I clinical trial (NCT04475523) is registered, in which the anti-VISTA monoclonal antibody CI-8993 is used as a therapeutic agent for solid tumors. Previously, a phase I clinical trial using CI-8993, called VSTB112 or JNJ-61610588, was terminated (NCT02671955) [176]. In addition, there is evidence from a clinical trial that small-molecule AUPM-170 or CA-170 inhibits VISTA and PD-L1/PD-L2 [177]. This is the first oral IC inhibitor approved for clinical trials [169]. CA-170 therapy in a phase I clinical trial (NCT02812875) was performed in a cohort of patients with advanced solid tumors that were resistant to or progressive with available therapeutic approaches. According to the Solid Tumor Response Evaluation Criteria (RECIST), 33 out of 50 cases showed stable disease. Partial or complete response was not achieved. Severe (grade 3 and 4) treatment-related side effects were observed in five patients [178].

Therefore, the data from the VISTA study are mixed. The relationship of VISTA expression with the clinical characteristics of the tumor is contradictory, and the results of antitumor activity in the case of VISTA inhibition are inconsistent. Such results are also characteristic of some other immune checkpoints that function under conditions of multiple interactions [179]. A deeper understanding of VISTA’s mechanisms of action in cancer is needed for the successful therapeutic application of VISTA in cancer immunotherapy.

### 2.5. BTLA

BTLA or CD272 is a transmembrane receptor expressed by naive T-lymphocytes, B-cells, macrophages, DCs, and natural killer T-cells (NKT) [180,181]. BTLA is involved in the regulation of immune cell homeostasis by inhibiting proliferation, the activation of B- and T-cells, and the production of cytokines [182]. In particular, BTLA negatively regulates the expansion and function of γδ T-cells [183], various subtypes of which both contribute to the progression of cancer and have antitumor activity [184]. A soluble form of the BTLA protein (sBTLA) is described as a potential prognostic and predictive marker in patients with clear cell renal cell carcinoma, pancreatic adenocarcinoma, and prostate cancer [185,186,187].

A recent study in patients treated with immune checkpoint (ICT) inhibitors for solid tumors found an association between serum levels of soluble BTLA (sBTLA) and median overall survival [188].

#### 2.5.1. Interaction with Ligands

A protein from the tumor necrosis factor receptor family, HVEM (herpes virus entry mediator), a product of the TNFRSF14 gene, is expressed by epithelial, endothelial, and hematopoietic cells and neurons. For HVEM, an interaction was shown with five molecules: BTLA, CD160, SALM5 LIGHT, and LTα. The co-inhibitory receptors BTLA and CD160 have similar affinity for HVEM, but CD160 has a longer dissociation time [189]. It has also been shown that BTLA and CD160 interact with an HVEM domain that is topologically different from those of other HVEM ligands [190]. After binding of the BTLA/HVEM pair in T-cells, processes resulting in the inhibition of signaling from TCR and CD28 are induced, while BTLA recruits SHP1 phosphatase to a greater extent. SHP1 has greater activity than SHP2, which is involved in PD-1-mediated inhibition [191]. There is also an assumption about the existence of an alternative mechanism for regulating the functions of T-cells, independent of SHP1/2 phosphatases [192]. At the same time, the NF-κB stimulatory signaling pathway, which is involved in maintaining T-cell tolerance, is activated in cells expressing HVEM [193,194]. T-cell activation is observed as a result of HVEM suppression in ovarian cancer cells and in an esophageal squamous cell carcinoma (ESCC) cell line [195,196]. An increased frequency of mutational changes in the TNFRSF14 gene is observed in lymphomas [196], melanoma, and colon adenocarcinoma [190]. Stedy et al. showed a tenfold increase in the affinity for BTLA of the obtained mutant form of HVEM and the lack of ability to interact with other ligands [197]. Thus, the mechanism of regulation of immune signals mediated by the BTLA:HVEM interaction is extremely unstable, which is especially important in the context of carcinogenesis.

HVEM expression is associated with a decrease in the amount of TILs and with a poor prognosis in ESCC and CRC, including patients with colorectal cancer metastases to the liver and other oncological diseases [196,198,199,200,201]. The expression of BTLA, in cases of follicular lymphoma (FL), is associated with a favorable prognosis. At the same time, high expression of HVEM is associated with an increased risk of transformation, while transformed FL is characterized by a low level of BTLA expression and a high level of HVEM [202]. In gastric cancer, there is a high frequency of overexpression of BTLA and HVEM, which is associated with a poor clinical prognosis [203].

To develop effective therapies, it is necessary to further study the processes of modulation of immune cell functions caused by BTLA/HVEM interaction in specific oncological diseases.

#### 2.5.2. Expression in Cancer

BTLA overexpression in CD8+ and CD4+ T-cells in gallbladder cancer is associated with poor survival [204]. In NSCLC, a high expression of protein products of the TNFRSF14 gene was found among patients with late stages of the disease and lymphatic invasions. In addition, the overexpression in tumor cells of BTLA or BTLA together with PD-L1 is associated with a decrease in relapse-free and overall survival [205]. Among patients with skin melanoma, a potential predictive value of the level of BTLA expression in relation to the effectiveness of immunotherapy with antibodies targeting PD-1 (Nivolumab, Pembrolizumab) and MAGE-3 was revealed [206]. In chronic lymphocytic leukemia, the aggressiveness and neglect of the disease is also associated with the level of sBTLA in the blood serum of patients [207]. In epithelial OC, BTLA expression was significantly correlated with TNM staging, lymph node metastasis, and recurrence (*p* < 0.05) [208]. At the same time, in colorectal cancer, high BTLA expression is associated with better survival and a lower level of metastasis to lymph nodes [209]. BTLA expression on TIL in adaptive cell therapy for melanoma is associated with a better response to treatment [210]. An interesting study was published by Kuncewicz et al., in which the peptides gD(1–36) (K10C–D30C) and gD(1–36) (A12C–L25C) were created and tested. They blocked the HVEM/BTLA interaction, while at the same time not preventing the formation of the HVEM complex with the alternative costimulatory ligand LIGHT [211].

**Table 1:** Clinical significance and results of preclinical studies of ICs and their ligands.

Receptor	Results of Preclinical Stydies	Ligands	Clinical Significance/ Results of Preclinical Stydies
TIM-3	The use of mAbs against TIM-3 stimulates the production of IFNγ. The antitumor efficacy of anti-TIM-3 is associated with the ratio of CD8+:CD4+ T-cells in the TILs pool. The combined use of mAbs targeting TIM-3, PD-1, and CTLA-4 has been shown to be more effective and well tolerated [47]. In models of lung adenocarcinoma, it was found that the use of mAbs targeting PD-1 can increase the expression of TIM-3. The effectiveness of the use of TIM-3 in overcoming resistance to therapy with mAbs targeting PD-1 has been shown [48]. The expression of LAG-3 and CTLA-4 was increased on CD8+ T-lymphocytes bound by the used mAbs targeting TIM-3 and PD-1. The combined use of mAbs targeting TIM-3 and CTLA-4 shows a synergistic effect in models [49].	Phosphotidylserine	-
		Galectin-9	Resistance to anti-PD-1 therapy has been observed in the presence of TIM-3+ lymphocytes and galectin-9-expressing MDSC [17]. The co-expression of galectin-9 and TIM-3 has been detected in various types of cancer [14,19]. The correlation of galectin-9 expression with better OS (in HCC and CRC) or PFS (in GC and NSCLC) has been shown [20]. The opposite data are available [16].
		Alarmin-1 (HMGB1)	HMGB1 is associated with progression and metastasis in NSCLC and CRC [25,26].
		CEACAM1	A synergistic antitumor effect has been shown with the simultaneous blockade of TIM-3 and CEACAM1, as well as CEACAM1 and PD-L1, on CRC models [36]. In the early stages of CRC, CEACAM1 inhibits tumor cell proliferation [34]. However, CEACAM1 is a diagnostic and prognostic marker in melanoma, and CEACAM1 is found in tumor samples and sera from patients with PC and is overexpressed in advanced stages of CRC, NSCLC, and other cancers [39].
LAG-3	It has been shown that the therapeutic use of PD-1 leads to an increase in the expression level of LAG-3 [80]. In NSCLC, the co-expression of LAG-3 and PD-1 on TILs and PD-L1 on tumor cells is shown [81]. A synergistic effect was observed from the combined use of mAbs binds LAG-3 and PD-1 in various tumor models [82].	MHC class II	MHCII is associated with survival, increased numbers of CD4+ and CD8+ T -cells in the TILs, and a good response to anti-PD-1 and PD-L1 immunotherapy in some cancers [65].
		FGL-1	The FGL-1/LAG-3 interaction blockade stimulates tumor immunity [67]. The reduced expression of FGL-1 increases the efficiency of CD8+ T-cell activation during LAG-3 blockade [69].
		Galectin-3	The restoration of cytolytic functions of CD8+ T- cells in response to the inhibition of galectin-3 was shown, which indicates the role of galectin-3 in the suppression of antitumor immunity. The direct involvement of galectin-3 in the processes of metastasis was revealed [72,73,74], as well as the association of galectin-3 expression with poor clinical prognosis [75]. However, in melanoma and glioblastoma, the presence of galectin-3 is beneficial for patients [76].
		LSECtin	A high level of soluble LSECtin in the blood serum of patients with CRC is associated with the presence of liver metastases [78]. The expression of LSECtin and its interaction with LAG-3 molecules are shown on B16 melanoma cells. It is accompanied by the suppression of the T-cell antitumor response, and the blockade of LSECtin/LAG-3 interaction restores the secretion of IFNγ [79].
TIGIT	The blockade of TIGIT has been shown to prevent the depletion of NK cells and stimulate NK-mediated tumor immunity, activate antitumor T-cell immunity, and promote the formation of immune memory [122,129]. The co-inhibition of TIGIT and PD-1 or PD-L1 with mAbs exhibited a significant therapeutic effect, up to the complete elimination of tumors [122,124,125,126]. Using mAb against TIGIT showed: restoration of the functions of effector T-cells; the induction of cellular cytotoxicity against regulatory T-cells; a direct cytotoxic effect on TIGIT+ tumor cells [127,130]. The high efficiency of the combined inhibition of PD-1 and CD96 or TIGIT and CD96 has been shown [128].	Nectin-2 (CD112)	Interaction with TIGIT leads to the corresponding transmission of inhibitory signals to immune cells. Nectin-2 is expressed in breast and ovarian tumors [111].
		Nectin-4 (PVRL4)	Nectin-4 blocking Abs stimulates an NK-mediated antitumor response [113]. The participation of nectin-4 in the processes of proliferation, invasion, and metastasis through the activation of Pi3k/Akt and WNT/β-catenin signaling pathways has been shown [114]. The revealed hyperexpression of nectin-4 by tumor tissues is associated with tumor aggressiveness and poor clinical prognosis [115,116].
		PVR (CD155)	Overexpression and the presence of a soluble form of CD155 in the blood serum of patients are associated with a poor clinical prognosis [102,106,107]. The association of the co-expression of TIGIT and CD155 with an unfavorable disease course in lung adenocarcinoma and primary SCC of the esophagus has been shown [106,108].
VISTA	In response to blocking VISTA with the use of mAbs, an increase in the number of TILs and the restoration of the functions of CD8+ T-cells were observed [147]. An increase in the expression of chemokines (CXCL9/10, CCL4/5) as well as cytokines (IFNβ, IL6, IL12, IL23, IL27, TNFα) was observed in tumor tissues [146]. However, the effective suppression of tumor growth was observed only when anti-VISTA mAbs was used in combination with anti-PD-1 mAbs [171,173] or CTLA-4 [170]. The blockade of VISTA caused an increase in tumor infiltration by immune cells and a decrease in the number of myeloid suppressor cells (MSCs). The therapeutic effect of anti-VISTA antibodies has been demonstrated in OC models highly expressing VISTA [143].	VSIG-3 (IGSF11)	The expression of VSIG-3 by tumor tissues was found in CRC, HCC, and in intestinal-type GC [151]. The overexpression of VSIG-3 is associated with the expression of VISTA, as well as with PD-L1 and PD-1, with a high degree of tumor malignancy, and a poor clinical prognosis in glioblastoma has been revealed [152]. Experimental models show the antitumor efficacy of the SG7 Ab, which inhibits VISTA binding to VSIG-3 and PSGL-1 [174].
		PSGL-1	The ability of PSGL-1 to bind to VISTA was shown at acidic values of the medium (pH 6.0). At lower pH values, an enhanced inhibitory effect of VISTA was shown, and the use of Abs capable of blocking the VISTA/PSGL-1 interaction restored the proliferative and secretory functions of T-cells [153]. Experimental models show the antitumor efficacy of the SG7 Ab, which inhibits VISTA binding to VSIG-3 and PSGL-1 [174].
		Galectin-9	The study of samples from patients with peritoneal carcinomatosis showed a high level of expression of galectin-9, VISTA and TIM-3 depleted TILs [156].
BTLA	The antitumor efficacy of anti-BTLA mAbs has been shown [212,213]. In the blockade of BTLA, an increase in the proliferation and expansion of NY-ESO-1-specific CD8+ T-cells was observed, and an increased efficiency of the use of mAbs targeting BTLA in combination with anti-PD-1 and anti-Tim-3 in melanoma was shown [214]. An increase in median OS [215], as well as the enhancing T-cell proliferation and cytokine production, was observed with the combination of anti-BTLA and anti-PD-1 therapies [216].	HVEM (TNFRSF14)	T-cell activation is observed as a result of HVEM suppression in OC cells and in the ESCC cell line [195,196]. HVEM expression is associated with a decrease in the number of TILs and with a poor prognosis in ESCC and CRC, including in patients with CRC metastases to the liver and other oncological diseases [196,198,199,200,201]. The high expression of HVEM is associated with an increased risk of transformation, while transformed FL is characterized by a low level of BTLA expression and a high level of HVEM [202]. In GC, an overexpression of BTLA and HVEM is associated with a poor clinical prognosis [203].

**Table 2:** Summary of ongoing clinical trials of receptor inhibitors.

Target	Drug	Number of Current Trials/ Phase	Type of Tumor	Some Published Results of Clinical Trials	
				Trial	Clinical Safety and Efficacy
TIM-3	Sabatolimab (MBG453)	16 I, II, III	Advanced or metastatic solid tumors Bone marrow diseases Glioblastoma Hematologic malignancies	NCT02608268 Phase I-Ib/II	Patients received sabatolimab (*n* = 133) or sabatolimab plus spartalizumab (*n* = 86). The MTD was not reached. No responses were seen with sabatolimab. Five patients receiving combination treatment had PR (6%; lasting 12–27 months) [51]
	TSR-022	4 I, II	Advanced or metastatic solid tumors Melanoma	NCT02817633 Phase I	In the group of 20 patients who received the TSR-022+TSR-042 combination, the ORR was 15% (3/20), and disease stabilization reached 40% (8/20) [52].
	LY3321367	1 I	Solid tumors	NCT03099109 Phase I	No DLTs were observed in the monotherapy (*n* = 30) or combination (*n* = 28) therapy. LY3321367 TRAEs occurred in ≥2 patients. In the NSCLC monotherapy expansion cohort, outcomes varied: anti-PD-1/L1 refractory patients [N = 23, ORR 0%, DCR 35%, PFS 1.9 months] versus anti-PD-1/L1 responders (*n* = 14, ORR 7%, DCR 50%, PFS 7.3 months). In combination expansion cohorts (*n* = 91), ORR and DCR were 4% and 42% [53]
	LY3415244, BsAb for PD-L1/TIM-3	1 I	Advanced solid tumors	NCT03752177 Phase Ia/Ib	Two patients (16.7%) developed clinically significant anaphylactic infusion-related reactions. One patient with PD-1 refractory NSCLC had a near partial response (−29.6%) [54]
	INCAGN02390	5 I	Solid tumors Melanoma	-	-
	BGB-A425	1 I	Advanced or metastatic solid tumors	-	-
	BMS-986258	1 I	Advanced cancer	-	-
	SHR-1702	2 I	Hematologic malignancies Advanced solid tumors	-	-
	RO7121661, BsAb for PD-1/TIM-3	2 I, II	Advanced or metastatic solid tumors Melanoma	-	-
LAG-3	Eftilagimod alpha (IMP321)	14 I, II	Advanced or metastatic solid tumors Melanoma	NCT00732082 Phase I	None of the 6 patients received 0.5 mg IMP321 experienced TRAEs. Of the 5 patients who received IMP321 at the 2 mg dose level, 1 experienced rash, 1 reported hot flashes, and 2 had mild pain at the injection sites [85]
				NCT00349934 Phase I	Thirty patients received IMP321 in three cohorts (doses: 0.25, 1.25 and 6.25 mg). Clinical benefit was observed for 90% of patients with only 3 progressors at 6 months. Additionally, t he ORR of 50% compared favorably to the 25% rate reported in the historical control group [86].
	Favezelimab (MK-4280)	10 I, II, III	Advanced or metastatic solid tumors Hematologic malignancies Melanoma	NCT03598608 Phase I/II	Fifteen patients received MK-4280 with pembrolizumab, four of whom achieved a partial response [87]
	Relatlimab (BMS-986016)	31 I, II	Advanced or metastatic solid tumors Hematologic malignancies Melanoma	NCT01968109 Phase I/IIa	Patients received relatlimab + nivolumab. In 61 efficacy-evaluable patients, ORR was 11.5% (1 complete, 6 partial (1 unconfirmed) responses); DCR was 49%. Median DOR was not reached (min [0.1þ], max [39.3þ]). ORR was 3.5-fold higher in patients with LAG-3 expression, 1% vs. <1%, regardless of PD-L1 expression. TRAEs occurred in 41% (gr 3/4, 4.4%; DC, 1.5%) [89]
				NCT03470922 Phase II	The median PFS was 10.1 months (95% confidence interval [CI], 6.4 to 15.7) with relatlimab–nivolumab as compared with 4.6 months (95% CI, 3.4 to 5.6) with nivolumab (hazard ratio for progression or death, 0.75 [95% CI, 0.62 to 0.92]; *p* = 0.006 by the log-rank test). PFS at 12 months was 47.7% (95% CI, 41.8 to 53.2) with relatlimab–nivolumab as compared with 36.0% (95% CI, 30.5 to 41.6) with nivolumab. Grade 3 or 4 TRAEs occurred in 18.9% of patients in the relatlimab–nivolumab group and in 9.7% of patients in the nivolumab group [90].
	TSR-033	2 I	Advanced solid tumors	-	-
	REGN3767	5 I, II, III	Advanced solid tumors	-	-
	Ieramilimab (LAG525)	5 I, II	Advanced solid tumors Hematologic malignancies Melanoma	NCT02460224 Phase I/II	Patients received fermilab (*n* = 134) or fermilab + spartalizumab (*n* = 121). Four patients experienced DLT in each treatment arm. No MTD was reached. TRAEs occurred in 75 (56%) and 84 (69%) patients in the single-agent and combination arms, respectively. Seven patients experienced SAEs in the single-agent (5%) and combination groups (5.8%). Antitumor activity was observed in the combination arm, with 3 (2%) CR and 10 (8%) PR. In the combination arm, 8 patients (6.6%) experienced SD for 6 months or longer versus 6 patients (4.5%) in the single-agent arm [93]
	FS118, BsAb for LAG-3/PD-L1	1 I, II	Advanced solid tumors Hematologic malignancies Melanoma	-	-
	RO7247669, BsAb for LAG-3/PD-1	5 I, II	Advanced or metastatic solid tumors Melanoma	-	-
TIGIT	Vibostolimab (MK-7684)	15 I, II, III	Advanced or metastatic solid tumors Melanoma Hematologic malignancies	NCT02964013 Phase I	Part A: 56% of patients receiving monotherapy and 62% receiving a combination of vibostolimab with pembrolizumab had TRAEs. Grade 3–4 TRAEs occurred in 9% and 17% of patients, respectively. No DLT was reported. The confirmed ORR was 0% for monotherapy and 7% for combination therapy. Part B: 39 patients had anti-PD-1/PD-L1-naive NSCLC, and all received combination therapy. TRAEs occurred in 85% of patients. The confirmed ORR was 26%, with responses observed in both PD-L1-positive and PD-L1-negative tumors. Sixty-seven had anti-PD-1/PD-L1-refractory NSCLC, and 56% receiving monotherapy and 70% receiving combination therapy had TRAEs. The confirmed ORR was 3% for monotherapy and 3% for combination therapy [131]
	BMS-986207	4 I, II	Advanced solid tumors Multiple myeloma	-	-
	Etigilimab (OMP-313M32)	2 I, II	Advanced or metastatic solid tumors	NCT03119428 Phase Ia/Ib	Thirty-three patients were enrolled (Phase Ia, *n* = 23; Phase Ib, *n* = 10). There was no DLT. MTD was not determined. Six patients experienced grade ≥ 3 TRAEs. In Phase Ia, 7 patients (30.0%) had stable disease. In Phase Ib, 1 patient had a PR; 1 patient had prolonged SD of nearly 8 months. Median PFS was 56.0 days (Phase Ia) and 57.5 days (Phase Ib) [133]
	Tiragolumab	38 I, II, III	Advanced or metastatic solid tumors Melanoma Hematologic malignancies	NCT02864992 Phase II	The RR by independent review was 46% (95% CI, 36 to 57), with a median DoR of 11.1 months (95% CI, 7.2 to could not be estimated) in the combined-biopsy group. The RR was 48% (95% CI, 36 to 61) among 66 patients in the liquid-biopsy group and 50% (95% CI, 37 to 63) among 60 patients in the tissue-biopsy group; 27 patients had positive results according to both methods. The investigator-assessed RR was 56% (95% CI, 45 to 66). TRAEs of grade ≥ 3 were reported in 28% [134]
				NCT03563716 Phase II	Patients were randomly assigned to receive tiragolumab + atezolizumab (67 (50%)) or placebo + atezolizumab (68 (50%)). After a median follow-up of 5.9 months (4.6–7.6, in the intention-to-treat population, 21 patients (31.3% [95% CI 19.5–43.2]) in the tiragolumab + atezolizumab group versus 11 patients (16.2% [6.7–25.7]) in the placebo + atezolizumab group had an objective response (*p* = 0.031). Median PFS was 5.4 months (95% CI 4.2-not estimable) in the tiragolumab + atezolizumab group versus 3·6 months (2.7–4.4) in the placebo + atezolizumab group (stratified hazard ratio 0.57 [95% CI 0.37–0.90], *p* = 0.015). Fourteen (21%) patients receiving tiragolumab + atezolizumab and 12 (18%) patients receiving placebo + atezolizumab had SAEs [135]
	Domvanalimab (AB154)	9 I, II, III	Advanced or metastatic solid tumors Melanoma Glioblastoma	-	-
	ASP8374	3 I	Advanced solid tumors Glioblastoma	-	-
VISTA	CI-8993	1 I	Solid tumors	-	-
	CA-170, VISTA/PD-L1/2 antagonist	2 I, II	Advanced or metastatic solid tumors lymphomas	NCT02812875 Phase I	According to the RECIST, 33 out of 50 patients who received CA-170 showed SD. PR or CR was not achieved. Severe (grade 3 and 4) TRAEs were observed in 5 patients. No DLTs were observed [171].
	JNJ-61610588	1 I	Advanced or metastatic solid tumors	-	-
BTLA	TAB004/JS004	7 I, II	Recurrent/ refractory malignant lymphoma Advanced or metastatic solid tumors	-	-

#### 2.5.3. Preclinical Studies

In vivo models show a high antitumor efficacy of the combination of anti-BTLA antibodies with chemotherapy (NCT00854399) [212]. In vivo breast carcinoma models have shown the effectiveness of antibodies against BTLA in controlling tumor growth and metastasis [213], accompanied by an increase in the number of NKT cells and the expression of cytotoxicity marker genes. In the blockade of BTLA, an increase in the proliferation and expansion of NY-ESO-1-specific CD8+ T-cells was observed, and an increased efficiency of the use of antibodies targeting BTLA in combination with anti-PD-1 and anti-Tim-3 in melanoma was shown [214]. In in vivo models of glioblastoma, an increase in median overall survival was observed with the combination of anti-BTLA and anti-PD-1 therapies [215]. The synergistic effect of this antibody combination in enhancing T-cell proliferation and cytokine production has been described in urothelial carcinoma [216].

#### 2.5.4. Clinical Trials

The TAB004/JS004 recombinant BTLA-specific antibody is currently approved by the FDA for clinical trials [217] and is being tested as a therapeutic agent for solid tumors and lymphomas in phase I and II clinical trials, alone or in combination with recombinant humanized anti-PD-1 monoclonal antibody (toripalimab, JS001) (NCT04137900, NCT04278859, NCT05000684, NCT04773951, NCT04929080, NCT04477772). Although BTLA has not yet been sufficiently studied, the available data indicate the promise of further study of this immune checkpoint as a target for activating the antitumor response.

## 3. Conclusions

An interesting feature of the considered IC receptors is the presence of a fairly wide range of molecules that interact with them and potentially act as their ligands. This situation gives rise to an ambiguous reaction of the immune system to environmental factors in the body. At the same time, the properties of these receptors as targets for the activation of the immune response that have been identified so far are not the same. The inhibition of LAG-3 is the most effective in terms of antitumor response. The expression of this receptor also shows the most unambiguous relationship with the clinical characteristics of cancer. This situation is not typical for all the considered receptors. The relationship of VISTA expression with the clinical characteristics of the tumor is contradictory. At the same time, the results of antitumor activity in the case of VISTA inhibition are also unstable. The situation is similar to that of TIM-3. We observed similar relationships when analyzing IC ligands—most often, those ligands that showed a good relationship with clinical characteristics were effective as targets for anticancer therapy [179]. In this regard, it should be noted that the expression of TIGIT and BTLA correlates well with clinical characteristics. In addition to the results of preclinical and clinical studies presented in this review, this finding indicates the high promise of TIGIT and BTLA as targets for anticancer therapy.

## Data Availability

The cited works can be found in the PubMed, PMC, Omicsonline, Clinicaltrial.gov, and Embase databases.
